# Oral metastasis as initial presentation of renal cell carcinoma: a case report with review of literature

**DOI:** 10.3332/ecancer.2025.1938

**Published:** 2025-07-02

**Authors:** Rukmini Bezbaruah, Asreen Suhana, Arpan Choudhury

**Affiliations:** 1 Department of Oncopathology, Dr. Bhubaneswar Borooah Cancer Institute, Guwahati 781016, India; 2 Department of Surgical Oncology, State Cancer Institute, Guwahati 781034, India

**Keywords:** renal cell carcinoma, head and neck, metastasis

## Abstract

Metastasis of renal cell carcinoma (RCC) to the head and neck region is rare. The metastases of RCC are radioresistant with surgery as the primary treatment modality. Here, we present a case of a 73-year-old male who presented with left facial swelling which on biopsy and immunohistochemistry showed metastatic RCC. The patient was re-evaluated again and the left renal primary was found out.

## Introduction

Tumour metastasis to the head and neck region comprises only 1% of oral malignant tumours [1] Renal cell carcinoma (RCC) is the third most common tumour to metastasise to the head and neck region, after lung and breast carcinoma [2]. Oral cavity metastasis is often late and often preceded by lung metastasis with poor prognosis [3]. The RCC metastases are radio-resistant and chemo-resistant with surgery being the only available treatment modality [4]. Surgery in such cases is often challenging due to the increased vascularity of the tumour and potentially being a late-stage phenomenon [5]. Here, we present a case of a 73-year-old man where metastasis to mandible led to the diagnosis of primary RCC.

## Case report

A 73-year-old male presented to the head and neck outpatient department with complaints of swelling on the left side of his face for last 6 months. Fine needle aspiration of the swelling was done in a local hospital where it was reported as Acinic cell carcinoma. The case was referred to our institute. The patient had no history of hematuria, cough or bone pain. There was no history of tobacco use or smoking.

**Figure 1.**
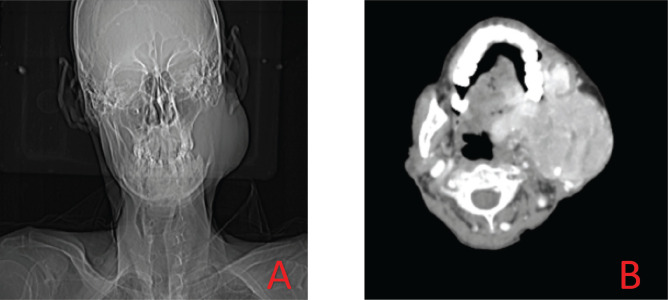
(a and b): CT images of tumour.

On local examination, left cheek swelling reaching upto zygoma was noted with free overlying skin. The mass was pedunculated and mobile measuring 7 × 5 cm^2^. The patient was advised to routine blood investigations along with contrast-enhanced computed tomography (CECT) neck and thorax. A biopsy from the lesion was taken. CECT [Figure 1(a and b)] showed a large nodular mass in left upper cervical region involving the left hemimandible. A significant number of vessels were noted in the periphery of the lesion.

**Figure 2.**
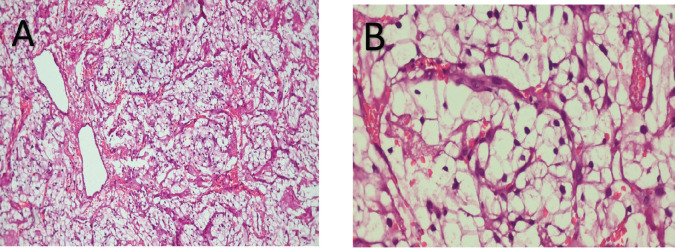
Low power (a) and high power (b) views of tumour with clear cell morphology.

On histopathological examination, sections showed nests and cords of clear cells separated by fibrovascular septae with no brisk mitosis or necrosis (Figure 2). The provisional diagnosis was given as malignant neoplasm with clear cell morphology. Considering the site and morphology, the following differential diagnoses were considered - Primary oral squamous cell carcinoma with clear cell change, Hyalinising clear cell carcinoma, Myoepithelial carcinoma, Mucoepidermoid carcinoma and metastatic clear cell RCC. Immunohistochemistry (IHC) was advised.

**Figure 3.**
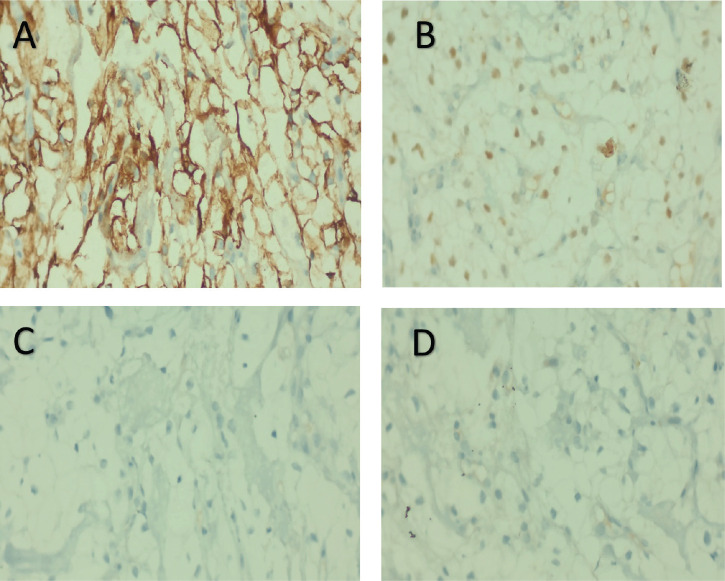
On IHC, tumour cells were positive for EMA (a), PAX8 (b), p40 (c) and CK7 (d).

The tumour cells were PAX8 and EMA positive, CK7, p63, S100 were negative (Figure 3). The final impression was given as metastatic carcinoma with a comment to rule out a renal primary.

The patient was re-evaluated and CECT abdomen showed left renal mass with bilateral pulmonary nodules and bony metastases. However, the patient was lost to follow-up. As the patient did not turn up for further treatment, a risk assessment could not be done. Based on the available hematological parameters (Hb, neutrophil, platelets and calcium), the risk assessment could not be done due to the unavailability of rest of the parameters (performance status and time of therapy).

## Discussion

Metastatic tumours to the oral cavity are extremely rare. Existing literature is based largely on sporadic case reports. Because of similar histological features, it is difficult to make a definitive diagnosis in many of these tumours when clear cells predominate [6]. Carcinomas from the kidney, liver, large bowel, prostate and thyroid can metastasise to the maxillofacial area and are known to have the potential for clear cell differentiation.

RCC metastatic lesions can sometimes mimic salivary gland and odontogenic tumours, but are characterised by hemorrhagic areas and the prominent sinusoidal vascular component [7].

IHC plays an important role in differentiating between malignant salivary gland tumours including clear cells and metastasis of RCC The major components of salivary gland tumours, which are characterised by clear cell changes, are contributed by the myoepithelial cells [8]. Based on this, primary salivary clear cell neoplasms can be divided into those that diagnostically require evidence of myoepithelial differentiation (myoepithelioma or myoepithelial carcinoma and epithelial myoepithelial carcinoma) and those that do not. Clear cell variants of salivary gland tumours, such as clear cell carcinoma, acinic cell carcinoma, mucoepidermoid carcinoma and oncocytoma, do not have myoepithelial differentiation. Table 1 shows the histological features useful for the differential diagnosis of salivary gland tumours containing malignant clear cells and metastatic RCC.

Schmidt *et al* [9] mentioned that the clinical usefulness of fine-needle aspiration cytology for the diagnosis of salivary gland lesions is controversial in their systematic review paper. Even in our case, the tumour was misdiagnosed outside as Acinic cell carcinoma.

The prognosis of patients with metastatic neoplasms in the oral region is poor with most patients dying within 1 year since metastatic tumours in the oral region are often preceded or accompanied by multiple metastatic lesions at other sites and organs [10].

Treatment of metastatic renal carcinoma to the head and neck is mainly palliative [11]. The use of systemic therapy in the setting of RCC metastasis to the oral cavity is limited. Therapeutic decisions are directed to minimise morbidity considering the poor long-term prognosis of the disease at such an advanced stage.

Newer agents targeting the VEGF pathway such as bevacizumab and sorafenib may provide hope for patients with metastatic RCC. Some trials have shown prolongation of progression-free survival with the use of these targeted molecular therapies in cytokine-refractory patients. Among 153 patients with oral metastasis of RCC described in the literature, surgery was performed in most patients (53 cases) and the local control rate was greater than 90%. In contrast, the local control rates of radiotherapy, pharmacotherapy and palliative surgery (debulking and cryo surgery) were relatively less. Therapeutic options for patients with metastatic RCC have expanded rapidly over the past decade with targeted immunotherapy being the new corner stone. Several emerging drugs are designed to enhance the antitumour immune response and are tested in ongoing trials. Combination therapies have revolutionised the first-line treatment of metastatic clear cell RCC. This started with the advent of dual immune checkpoint blockade using ipilimumab plus nivolumab. In the CheckMate-214 trial, ipilimumab plus nivolumab improved overall survival compared with sunitinib in patients with intermediate- and poor-risk disease according to the International Metastatic RCC Database Consortium [12]. This led to its U.S. Food and Drug Administration approval in 2018 as a first-line combination therapy option for patients with metastatic RCC.

## Conclusion

Although rare, it should be kept in mind that orofacial symptoms such as mandibular swelling can be the initial presentation of metastatic disease. Oral metastasis of RCC has a poor prognosis due to the presence of concurrent disseminated metastases. Surgery is recommended because of its high local control rate and good quality of life.

## Conflicts of interest

There were no conflicts of interest.

## Funding

This work was self-financed by the authors and received no external funding.

## Informed consent

Written informed consent was obtained from the patient for publication of this case.

## Author contributions

Dr Rukmini Bezbaruah, Dr Asreen Suhana and Dr. Arpan Choudhury contributed to the study conception and design.

Material preparation, data collection and analysis were performed by Dr Rukmini Bezbaruah and Dr. Asreen Suhana.

The first draft of the manuscript was written by Dr Rukmini Bezbaruah and all authors commented on the previous versions of the manuscript. All authors read and approved the final manuscript.

## Figures and Tables

**Table 1.** Diagnostic immunohistochemical markers and histopathological features for differential diagnosis of clear cell tumours in oral cavity.

Name	Histopathology	IHC
RCC	Highly vascular stroma, sinusoidal spaces	CD10(+), vimentin (+), PAX 8 (+)
Clear cell carcinoma	Hyalinizing stroma	Epithelial markers (+), Myoepithelial markers (−)
Acinic cell carcinoma	Granular cytoplasm	DOG 1
Mucoepidermoid carcinoma	Mucus and intermediate cells	Myoepithelial markers (−), p63(+)
Epithelial myoepithelial carcinoma	Biphasic tubular structure-outer clear and inner cuboidal cells	Epithelial markers (+), Myoepithelial markers (−)
